# The Field of Psychotherapy: Over 100 Years Old and Still an Infant Science

**DOI:** 10.32872/cpe.v2i1.2753

**Published:** 2020-03-31

**Authors:** Marvin R. Goldfried

**Affiliations:** a Stony Brook University, Stony Brook, NY, USA

**Keywords:** clinical trials, therapy alliance, clinical training, practice-research gap, psychotherapy integration, RDoC

Although the field of psychotherapy has been in existence for well over 100 years, we have not yet reached the point of becoming what sociologists of science have called a “mature” science. Sociologists who study the evolution of different scientific enterprises have defined a mature field as one where there is not only the *cutting edge*–where new contributions are being made–but also an agreed-upon *core or consensus*. Although there is often disagreement among those contributing to the cutting edge of a mature science, there nonetheless remains the agreed-upon core. In the field of psychotherapy, although there are clinicians and researchers who have been working at the cutting edge, what we lack is an agreed-upon core or consensus. In essence, even after more than 100 years, psychotherapy is still considered an infant science.

One of my first experiences in recognizing the disjointed nature of psychotherapy occurred when I was in graduate school way back in the 1950s when I was traumatized by Paul Meehl over diner. As I have described elsewhere:

Meehl paid a visit to our program, delivered a colloquium, and spent some time with us graduate students. I was fortunate enough to be among a small group of students that went out to dinner with him. This was a rare treat, especially since I had read virtually everything Meehl had written, and had enormous respect for his insights on research, practice, and the philosophy of science. Indeed, he was my role model. At one point during the evening, someone asked him the question about the extent to which his clinical work was informed by research. Without any hesitation, he replied, “Not at all.” As someone who was struggling to adopt the identity of scientist–practitioner, I left this memorable dinner disheartened. I don’t think I ever fully recovered. The challenge of how we could close the gap between research and practice has stayed with me all these years, and because I am attracted to challenges–my experiential colleagues would probably call it “unfinished business”–I have continued to be intrigued with the integration of research and practice. (Goldfried, 2015, pp. 1086-1087).

One can most assuredly forgive Meehl for not making use of research in his clinical work; there was relatively little research on psychotherapy in the 1950s. However, the gap between research and practice continues to exist, even though there is now an extraordinary amount of research on psychotherapy. However, the researchers complain that the clinicians are not making use of their findings, and the clinicians are complaining that the researchers are not studying issues that are relevant to their therapeutic practices. And although there are many professionals in the field who are trying to close this gap, it nonetheless continues to exist.

Another most significant factor that prevents the field of psychotherapy from forming a core is that we think in terms of schools of therapy rather than basic processes or principles. That the field of psychotherapy is made up of so many different schools of therapy also means that these views compete with each other. Although some therapists maintain that diversity is good, sociologists of science have characterized a field with competing schools of thought as being “immature.”

There are several factors that motivate the development of competing schools. Relatively little professional credit goes to those who simply repeat what has already been said in the past. After all, careers are made by *making* history, not *knowing* it. In the field of psychotherapy, there are also social, personal, and economic factors that operate as well. However, developing still another new school of therapy is working at the cutting edge, and does nothing to contribute to an agreed-upon core. In essence, the field of psychotherapy has been spinning its wheels by proliferating different approaches to therapy.

We seem to be more interested in what is “new” than what is “old.” Here again, what is new is at the cutting edge and therefore is more likely to be rewarded. When a new school of therapy is proposed, it often comes with its own set of theoretical jargon (e.g., “observing ego,” “metacognition,” “decentering,” “reflective functioning”). One of the unintended consequences of developing new terms for old phenomenon is that clinical and research contributions on a given topic may disappear by virtue of the fact that the keywords used to search the literature change. Thus, to talk about “values” as an important phenomenon in therapeutic intervention can mask earlier work on encouraging patients to identify and express their needs. Thus, a “new wave” of therapy that comes up with new terms for old phenomena may wash away relevant keywords, such as “assertiveness.”

Although I most certainly do not propose that I have *the* answer to how the field of psychotherapy might move forward, there nonetheless are directions I believe might be pursued (for further details please refer to Goldfried, 2019). I have no doubt that one day the field of psychotherapy will develop an agreed-upon core or consensus, using a common language that facilitates communication to all.
